# ﻿New species of the genus *Trichosetodes* Ulmer, 1915 (Trichoptera, Leptoceridae) from Ratanakiri province, Cambodia, based on morphological and molecular data

**DOI:** 10.3897/zookeys.1182.105716

**Published:** 2023-10-12

**Authors:** Pongsak Laudee, Hans Malicky, Chamroeun Kong, Masaki Takenaka, Koji Tojo

**Affiliations:** 1 Faculty of Innovative Agriculture and Fishery Establishment Project, Prince of Songkla University, Surat Thani Campus, Surat Thani, Muang District Province, 84100, Thailand Prince of Songkla University Surat Thani Thailand; 2 Sonnengasse 13, A-3293 Lunz am See, Austria Unaffiliated Lunz am See Austria; 3 Kampong Speu Institute of Technology, Amleang, Kampong Speu Province, Cambodia Kampong Speu Institute of Technology Amleang Cambodia; 4 Department of Biology, Faculty of Science, Shinshu University, Asahi 3-1-1, Matsumoto, Nagano 390-8621, Japan Shinshu University Matsumoto Japan

**Keywords:** Aquatic insects, biodiversity, caddisfly, Mekong River basin, morphology, ribosomal mRNA, taxonomy

## Abstract

Three new species of *Trichosetodes*, namely *T.carmelae***sp. nov.**, *T.katiengensis***sp. nov.** and *T.ratanakiriensis***sp. nov.** are described and illustrated by male specimens. The male genitalia of *T.carmelae***sp. nov.** can be distinguished from the other 16 species of the genus found in Southeast Asia by the shape of the phallicata. The phallicata of *T.carmelae***sp. nov.** bears a tuft of long hairs in the middle of the dorsal edge. *Trichosetodeskatiengensis***sp. nov.** can be distinguished from the other species in Southeast Asia by the shape of the phallicata which is divided into dorsal and ventral branches in lateral view, and *T.ratanakiriensis***sp. nov.** by the characters of the left inferior appendage and the shape of segment IX. The posterior end of the left inferior appendage of *T.ratanakiriensis***sp. nov.** is not forked and the ventral and lateral views of the posteroventral lobes of segment IX are rounded. Illustrations of male genitalia of *Trichosetodeskampongspeuensis* Malicky & Kong, 2020 are provided for comparison. The molecular diversity of new *Trichosetodes* species was analyzed using the mitochondrial large subunit ribosomal rRNA gene region (16S rRNA). In terms of their genetic divergence, *T.ratanakiriensis***sp. nov.** and *T.kampongspeuensis* exhibited remarkable proximity, with only a 1.4% distance. On the contrary, *T.carmelae***sp. nov.** displayed genetic disparity exceeding 6.3% when compared to both *T.ratanakiriensis***sp. nov.** and *T.kampongspeuensis*.

## ﻿Introduction

*Trichosetodes* Ulmer is a genus of Trichoptera in the family Leptoceridae, which can be identified by the crescent shape of the abdominal segment IX from the lateral view, finger-like preanal appendages and a tuft of long hairs anterodorsally on abdominal segment IX (Schmid 1987; Gibon 1991; Malicky 2006a; Malicky and Graf 2020). Fifty-five species have been described worldwide. However, while *Trichosetodes* spp. has been mainly found from Oriental regions, three and thirteen species have been reported from the East Palearctic and Afrotropical regions, respectively (Malicky 2010; Mey and De Moor 2019; Kimura and Kuranishi 2020; Laudee et al. 2020; Malicky and Graf 2020; Morse 2023). In Asia, *T.atisudhara* Schmid, 1987, *T.compositus* Martynov, 1936, and *T.pandrosus* Malicky, 2006 were reported from Nepal (Malicky 2006b), and *T.japonicus* Tsuda, 1942 from Japan and the Korean Peninsula (Kimura and Kuranishi 2020; Park and Kong 2020). Yang et al. (2016) reported that seven species of *Trichosetodes*, namely *T.bicornis* Yang & Morse, 2000, *T.falcatus* Yang & Morse, 2000, *T.insularis* Schmid, 1987, *T.lasiophyllus* Yang & Morse, 1989, *T.phylloideus* Yang & Morse, 2000, *T.rhamphodes* Yang & Morse, 2000 and *T.serrayus* Yang & Morse, 2000 were found from several parts of China. In India, thirteen species of *Trichosetodes* have been recorded from several areas (Saini et al. 2001). In Southeast Asia, Malicky (2010) reported that 12 species of *Trichosetodes* were collected from Thailand, Laos, Peninsular Malaysia, Sumatra (Indonesia) and Java (Indonesia). Oláh (2013) described two new species of *Trichosetodes*, namely *T.harmas* Oláh, 2013 and *T.sotet* Oláh, 2013 from Vietnam. In Myanmar, Malicky and Laudee (2017) described a new species of *Trichosetodes*, *T.asphor* Malicky & Laudee, 2017, which was found from Taninthayi Division. Recently, *T.kampongspeuensis* Malicky & Kong, 2020 in Laudee et al. (2020) from Cambodia was described.

Presented herein is a report encompassing selected findings derived from an extensive investigation into the caddisfly biodiversity within the Mekong River and its network of tributaries. This study led to the collection and subsequent description of three distinct *Trichosetodes* species originating from the Katieng Waterfall, situated within the confines of Cambodia’s Ratanakiri Province.

## ﻿Material and methods

Male adult caddisfly specimens were collected overnight with a UV pan light trap (12V, 10W) near Katieng Waterfall and its stream, in Ratanakiri Province, Cambodia. The adult Trichoptera specimens were collected and preserved in 70% ethanol. Adult male genitalia of the new species were excised and macerated in 10% KOH at 60 °C for 30–60 min. The male genitalia of the new species were drawn with pencil while using a compound microscope with a drawing tube, and then final vector-graphic illustrations were prepared from the pencil templates with Adobe Illustrator 2023 software.

The holotypes and some paratypes of the new species were stored in 70% ethanol and were deposited at the Princess Maha Chakri Sirindhorn Natural History Museum, Prince of Songkla University, Hat Yai Campus, Hat Yai District, Songkhla Province, Thailand (**PSUNHM**). Some paratypes are deposited in the collection of Hans Malicky, Lunz am See, Austria (**CHM**), the National Museum, Prague, Czech Republic (**NMPC**) and the Clemson University Arthropod Collection, Clemson, South Carolina, USA (**CUAC**). Terminology of structure of genitalia follows Yang and Morse (2000).

The DNA was extracted from the ethanol-preserved tissue of the specimens, and purified using the DNeasy Blood & Tissue Kit (Qiagen, Hilden, Germany), according to the manufacturer’s instructions. The region of mitochondrial DNA (mtDNA) coding 16S rRNA region was amplified by a polymerase chain reaction (PCR) using the primer set 16S rRNA: 5’- TRA CYG TRC AAA GGT AGC -3’ and 5’- CCG GTY TRA ACT CAR ATC ATG T -3’ (Takenaka et al. 2023). Regarding each reaction, 1.0 μL of 10× Ex Taq buffer, 0.8 μL dNTP mixture (included 25 mM MgCl_2_), 0.05 μL of 5 U/μL Ex Taq polymerase (TAKARA, Shiga), 0.25 μL of each primer and 2.0 μL of extracted DNA for in total 10 μL were applied. The PCR protocol was: 94 °C for 2 min; 35× (94 °C for 1 min, 50 °C for 1 min, 72 °C for 1 min); and 72 °C for 3 min. The PCR products were purified using ExoSAP-IT Express (Thermo Fisher Scientific K.K., Tokyo, Japan). Purified DNA fragments were sequenced directly by an automated method using a BigDye Terminator v.1.1 Cycle Sequencing Kit (Applied Biosystems, Foster City, CA, USA) on an automated DNA Sequencer (ABI 3130 or 3130xl DNA Analyzer; Perkin Elmer/Applied Biosystems).

All sequences obtained have been submitted to the DNA data bank of Japan (DDBJ database) (GenBank accession numbers: *Trichosetodescarmelae* sp. nov., C761851; *T.ratanakiriensis* sp. nov., LC761852; *T.kampongspeuensis*, LC761853). Regarding the outgroup, we included the DNA sequence data of *Setodesbrevicaudatus* Yang & Morse, 1989 (GenBank accession numbers: OL678050 and NC069285). Sequence alignment and editing were performed using MEGA v.7.0.26 (Kumar et al. 2016) and CLC Workbench software (CLC bio, Aarhus, Denmark). All sequence data were aligned using MAFFT v.7.222 (Katoh and Standley 2013). Phylogenetic analyses based on the mtDNA 16S rRNA (434 bp) were performed by the Neighbor-Joining (NJ) method using MEGA v.7.0.26 (Kumar et al. 2016) with 1000 bootstrap cycles. Genetic distances (*p*-distance) between the species were calculated using MEGA v.7.0.26 (Kumar et al. 2016).

## ﻿Systematics

### ﻿ Trichosetodes carmelae

Taxon classificationAnimaliaTrichopteraLeptoceridae

Laudee & Malicky sp. nov.

1D10BFF9-8636-5B86-B745-98934F7F1600

https://zoobank.org/78DA92FF-BAEB-4BD3-9097-AE4E453A4255

Fig. 1

#### Type material.

***Holotype*. Male.** Cambodia: Ratanakiri Province: Banlung, Katieng Waterfall, 13°40'38"N, 106°58'33"E, elev. 203 m, 13.v.2022, Pongsak Laudee. (PSUNHM). ***Paratypes***: Same data as holotype, 6 males: 2 males (PSUNHM), 2 males (CHM), 2 males (CUAC).

#### Diagnosis.

The male genitalia of the new species are moderately similar to those of *T.sotet* Oláh, 2013 described in Vietnam in the form of segment IX and inferior appendages, as well as of *T.insularis* Schmid, 1987 in the form of segment IX and segment X; but the phallicata or aedeagus is clearly different. The phallicata of *T.sotet* and *T.insularis* are divided into subbasodorsal branch and subbasoventral branch, whereas such features are missing in *T.carmelae*. The phallicata in *T.carmelae* bears tuft of long hairs in the middle of dorsal edge, which does not occur in *T.sotet*. The phallicata in *T.carmelae* is slightly bent upward subapically, while it is curved downward in *T.sotet*.

#### Description.

Length of each male forewing 4.5 mm (*N* = 5); specimens in alcohol with head, thorax, abdomen, legs, forewings light brown.

**Figure 1.**
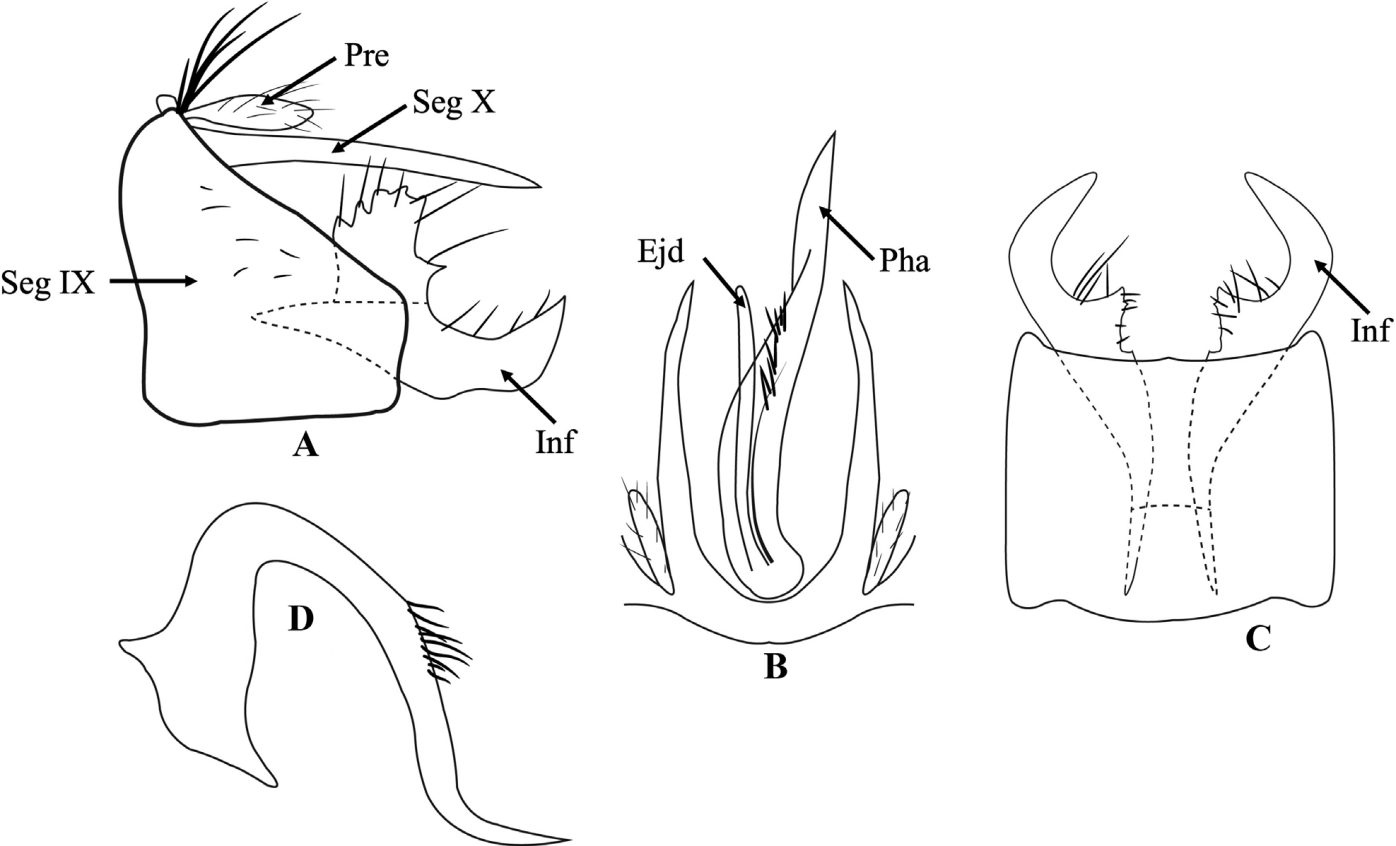
*Trichosetodescarmelae* sp. nov. male genitalia **A** segment IX and inferior appendages, left lateral **B** segment X and phallus, dorsal **C** segment IX and inferior appendages, ventral **D** phallus, left lateral. Abbreviations: Pre = preanal appendages (paired), Pha = phallicata, Seg IX = segment IX, Seg X = segment X, Inf = inferior appendage (paired), Ejd = ejaculatory duct.

Male genitalia (Fig. 1A–D). Segment IX right trapezoid, anterior margin convex dorsally and truncated ventrally, posterior margin truncated in lateral view (Fig. 1A); rectangular with pair of notches anteriorly in ventral view (Fig. 1C). Preanal appendages thumb-like covered with hairs (Fig. 1A, B). Segment X produced in pair of javelin-like processes each with acute apex (Fig. 1A, B). Inferior appendages each with dorsal and ventral lobes, dorsal lobe broad rectangular, serrated dorsally with small triangular process, ventral lobe slender with acute apex directed dorsad in lateral view (Fig. 1A); in ventral view, claw-like, bent inward, each with inner broad tooth sub-basally (Fig. 1C). phallicata in dorsal view, slender, lancet-like, acute apex, with isolated bunch of long hair in the middle; ejaculatory duct tube-like, about half as long as phallicata length (Fig. 1B); in lateral view, phallus typically large, axe-like, broad basally, strongly curved backward subbasally, with isolated bunch of long hairs dorsally, bent subapically, sharp apex (Fig. 1D).

#### Etymology.

The species name is dedicated to Dr Carmela R. Centrino who works for United Nations Industrial Development, Vienna International Centre for Southeast Asian Countries.

### ﻿ Trichosetodes katiengensis

Taxon classificationAnimaliaTrichopteraLeptoceridae

Laudee & Malicky sp. nov.

5AA2A611-A1AD-588A-A1BA-8FA849DBCF26

https://zoobank.org/A1F2CDD2-ABBA-4CDF-A5E9-87E5C3D3CDE3

Fig. 2

#### Type material.

***Holotype*. Male.** Cambodia: Ratanakiri Province: Banlung, Katieng Waterfall, 13°40'38"N, 106°58'33"E, elev. 203 m, 13.v.2022, Pongsak Laudee. (CHM). ***Paratypes***: same data as holotype. 2 males: 1 male (PSUNHM), 1 male (CHM).

#### Diagnosis.

The male genitalia of the new species are moderately similar to those of *Trichosetodespales* Malicky & Chaibu, 2006 described in Thailand, in the form of segment IX and inferior appendages, however, the phallus is clearly different. The phallicata of *T.pales* is divided into a dorsal branch, median branch and ventral branch, however, these features are missing in *T.katiengensis*. In addition, ventral lobes of inferior appendages are truncated and pointed in *T.pales* in both ventral and lateral views.

#### Description.

Length of each male forewing 3.5–4.0 mm (*N* = 3); specimens in alcohol with head, thorax, abdomen, legs, forewings dark brown.

**Figure 2.**
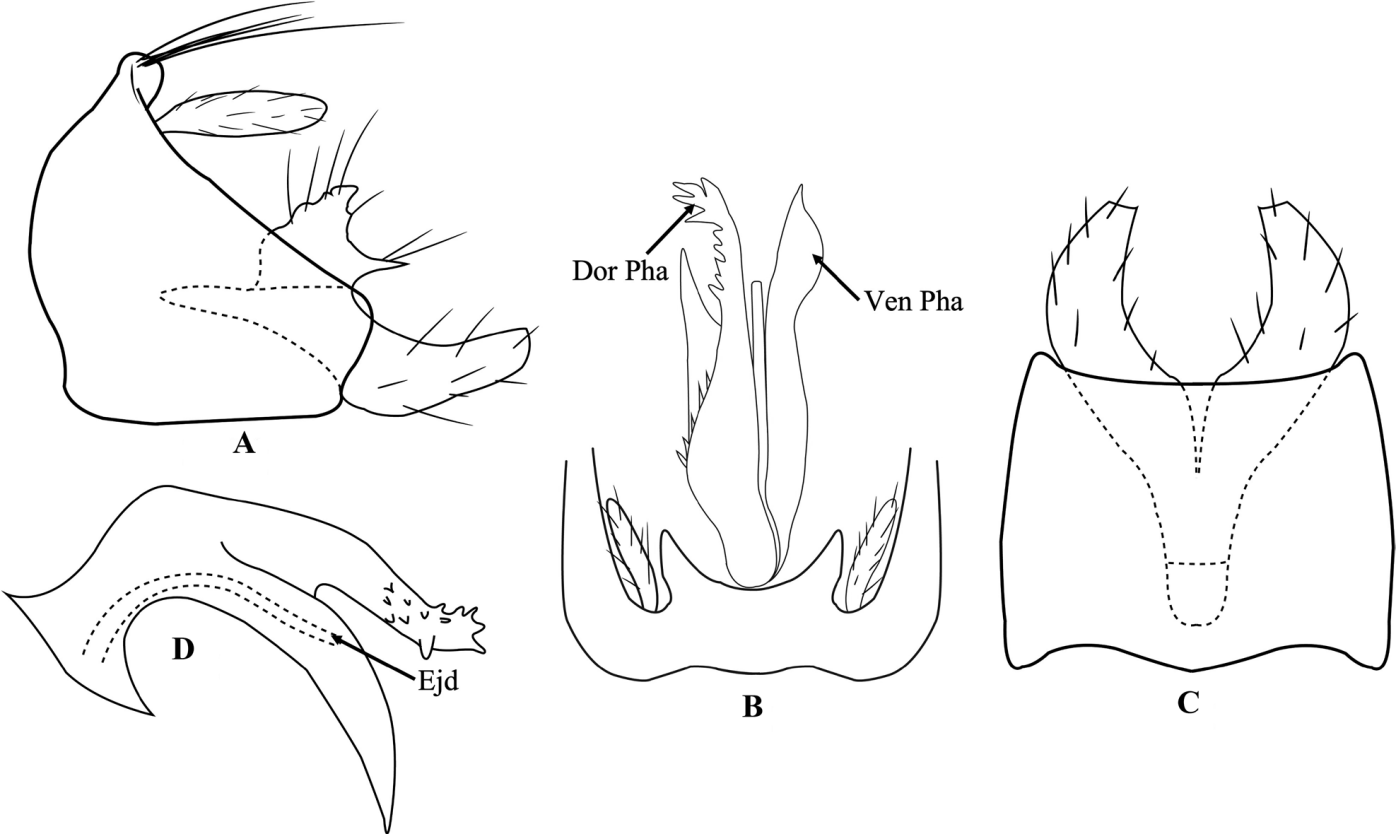
*Trichosetodeskatiengensis* sp. nov. male genitalia **A** segment IX and inferior appendages, left lateral **B** segment IX and phallus, dorsal **C** segment IX and inferior appendages, ventral **D** phallus. Abbreviations: Dor Pha = dorsal branch of phallicata, Ven Pha = ventral branch of phallicata, Ejd = ejaculatory duct.

Male genitalia (Fig. 2A–D). Segment IX with pair of thumb-like lobes posteriorly in dorsal view (Fig. 2B); right trapezoid, anterior margins convex with small lobe anterodorsally, posterior margin slightly truncated in lateral view (Fig. 2A); square with shallow notches anteriorly in ventral view (Fig. 2C). Preanal appendages thumb-like covered with hairs (Fig. 2A, B). Segment X not evident. Inferior appendages with dorsal and ventral lobes, dorsal lobe triangular in lateral view with sharp process basoposteriorly, ventral lobe tubular and truncated apically in lateral view (Fig. 2A); in ventral view, horn-like, bent inward, truncated apically (Fig. 2C). In dorsal view, phallus complex, phallicata divided into dorsal and ventral branches; dorsal branch with outer edge denticulated and ventral branch undulated with acute apex; ejaculatory duct short and thin (Fig. 2B). In lateral view, phallicata sickle-like with dorsal and ventral branches, dorsal branch of phallicata straight, covered with numerous short protrusions subapically, apex with small spines; ventral branch of phallicata claw-like, curved downward, acute apex; ejaculatory duct curved tube-like (Fig. 2D).

#### Etymology.

The species is named for the type locality, Katieng Waterfall.

### ﻿ Trichosetodes ratanakiriensis

Taxon classificationAnimaliaTrichopteraLeptoceridae

Laudee & Malicky sp. nov.

F36027AC-D7C4-5B55-9F2F-C7D8531DCB25

https://zoobank.org//873AF5A4-76F5-423D-8E48-4A45AD10BBB1

Fig. 3

#### Type material.

***Holotype*. Male.** Cambodia: Ratanakiri Province: Banlung, Katieng Waterfall, 13°40'38"N, 106°58'33"E, elev. 203 m, 13.v.2022, Pongsak Laudee. (CHM). ***Paratypes***: same data as holotype. 33 males: 18 males (PSUNHM), 5 males (CHM), 5 males (CUAC), 5 males (NMPC).

#### Diagnosis.

The male genitalia of *T.ratanakiriensis* are moderately similar to those of *T.pandareos* Malicky, 2006 described in Laos and *T.kampongspeuensis* Malicky & Kong, 2020 (Fig. 4), however it can be differentiated by the shape of left inferior appendage and the shape of segment IX. The left inferior appendage is forked in *T.pandareos* whereas this feature is missing in *T.ratanakiriensis*. In addition, the prolongations of the subapicoventral part of segment IX are symmetric in *T.pandareos* but asymmetric in *T.ratanakiriensis* in ventral view. Compared to *T.kampongspeuensis*, *T.ratanakiriensis* exhibits a longer, cylindrical right inferior appendage with a sharp apex in lateral view, whereas in *T.kampongspeuensis*, this appendage is oval and splits into two at the tip. The prolongations of subapical part of segment IX in ventral view are thin in *T.kampongspeuensis* but prominent in *T.ratanakiriensis*.

#### Description.

Length of each male forewing 5 mm (*N* = 12); specimens in alcohol with head, thorax, abdomen, legs, forewings dark brown.

**Figure 3.**
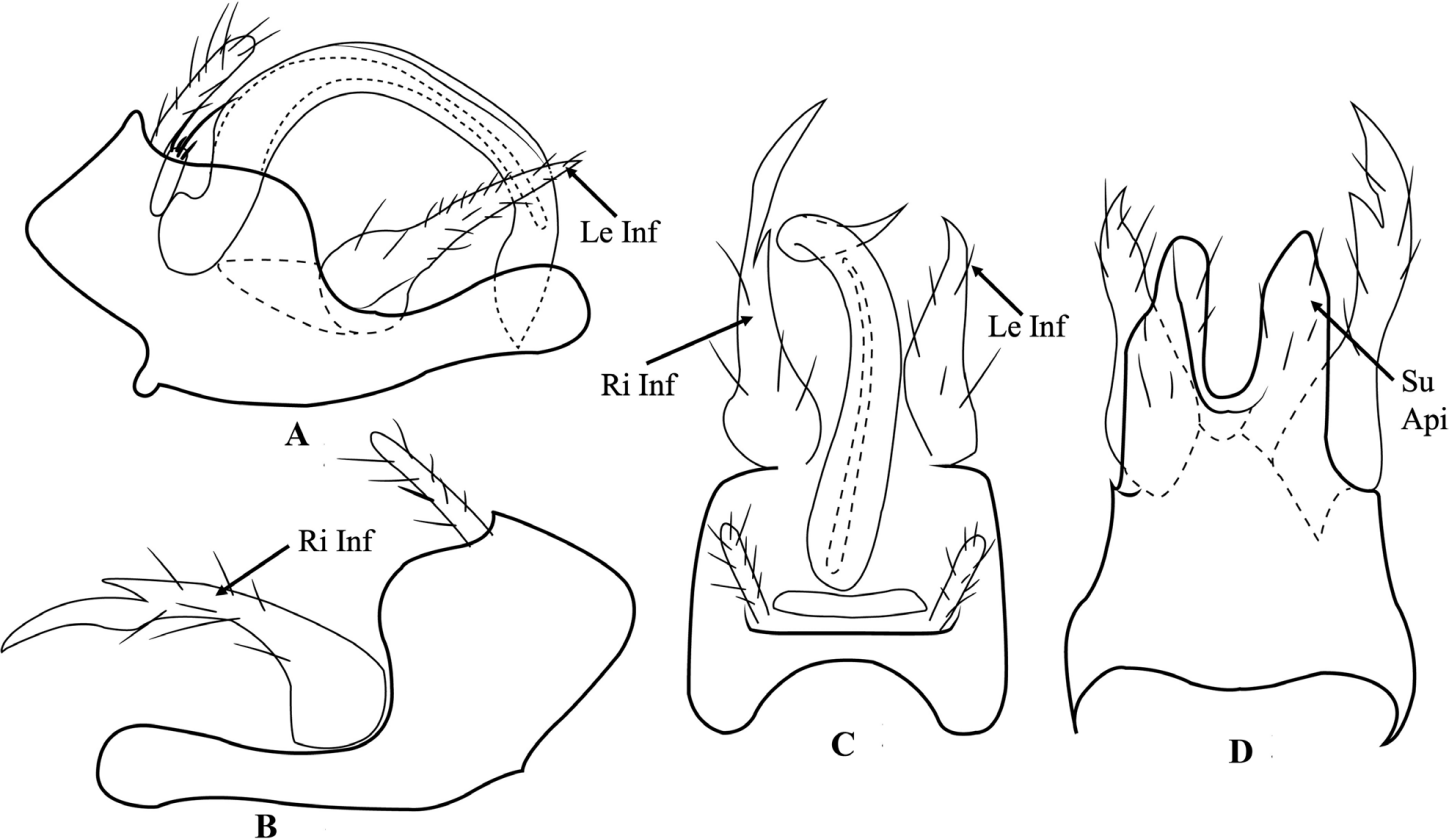
*Trichosetodesratanakiriensis* sp. nov. male genitalia **A** segment IX and inferior appendages, left lateral **B** segment IX and inferior appendages, right lateral **C** segment IX and phallus, dorsal **D** segment IX, ventral. Abbreviations: Le Inf = left inferior appendages, Ri Inf = right inferior appendage, Su Api = subspicoventral part of segment IX.

Male genitalia (Fig. 3A–D). Segment IX square with U-shaped incision anteriorly in dorsal view (Fig. 3C); in left lateral view, complicated shape, strongly convex anteriorly, undulated edge dorsally, curved downward ventrally with pimple sub-anteroventrally, long cylindrical subapicoventrally, rounded apically (Fig. 3A); in right lateral view the same as left lateral view but without pimple (Fig. 3B); in ventral view, vertical profile rectangular with 1/3 of height in U-shaped incision apically, rounded apex (Fig. 3D). Preanal appendages slender covered with hairs (Fig. 3A, B). Segment X not evident. Inferior appendages asymmetrical; circular basally, conical apically in left lateral view (Fig. 3A); in right lateral view, cylindrical with expanded basally, curved downward with tooth at dorsal edge subapically, pointed apically (Fig. 3B); in dorsal and ventral view, left inferior appendage claw-like, right inferior appendage long claw-like with an inner tooth. Phallicata large, tubular, curved and bent subapically, pointed apically in dorsal view; in lateral view, upside down U-shaped, pointed apically.

**Figure 4.**
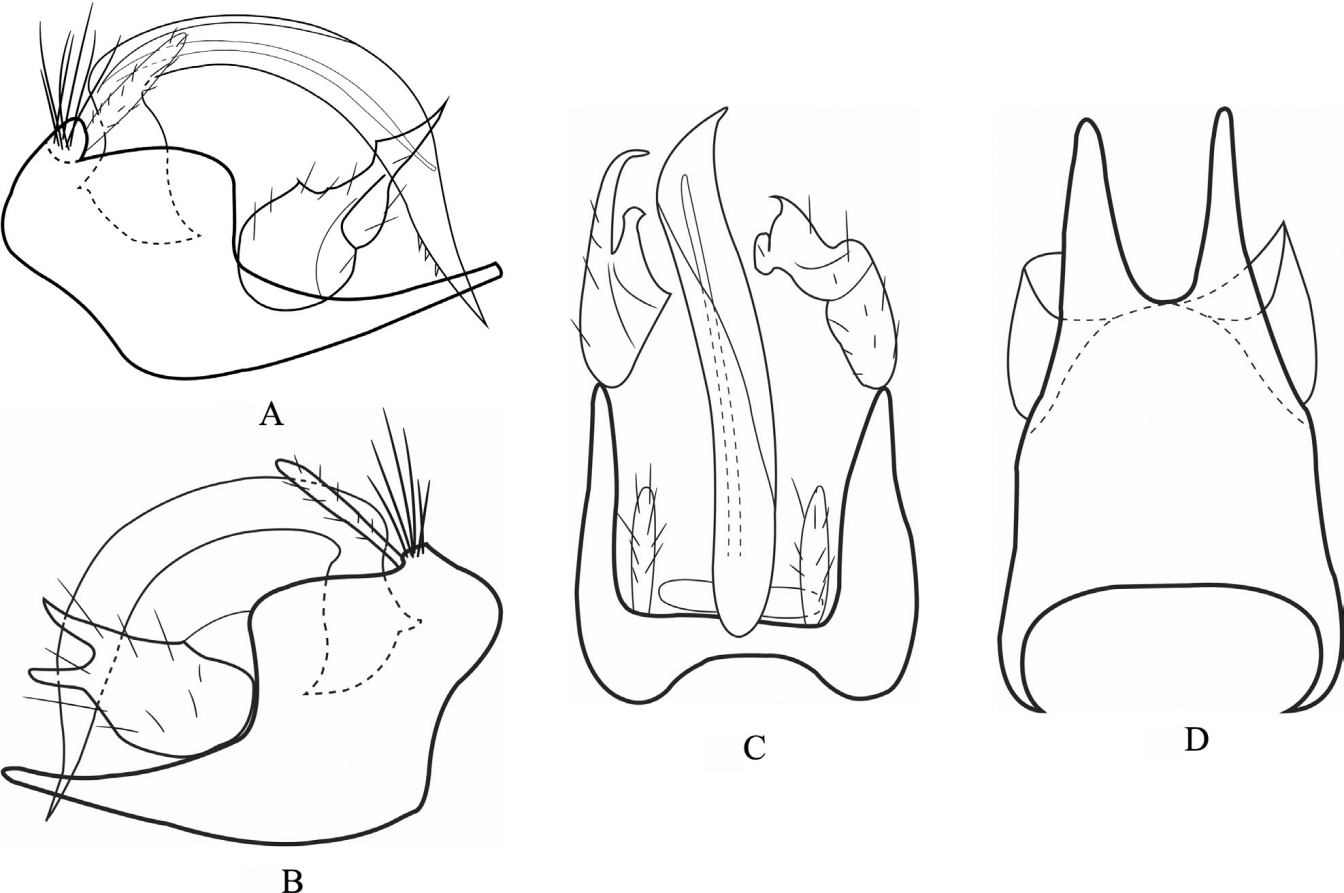
*Trichosetodeskampongspeuensis* male genitalia **A** segment IX and inferior appendages, left lateral **B** segment IX and inferior appendages, right lateral **C** segment IX and phallus, dorsal **D** segment IX, ventral.

#### Etymology.

The species is named for the type locality, Ratanakiri Province.

##### ﻿Molecular analysis

**Figure 5.**
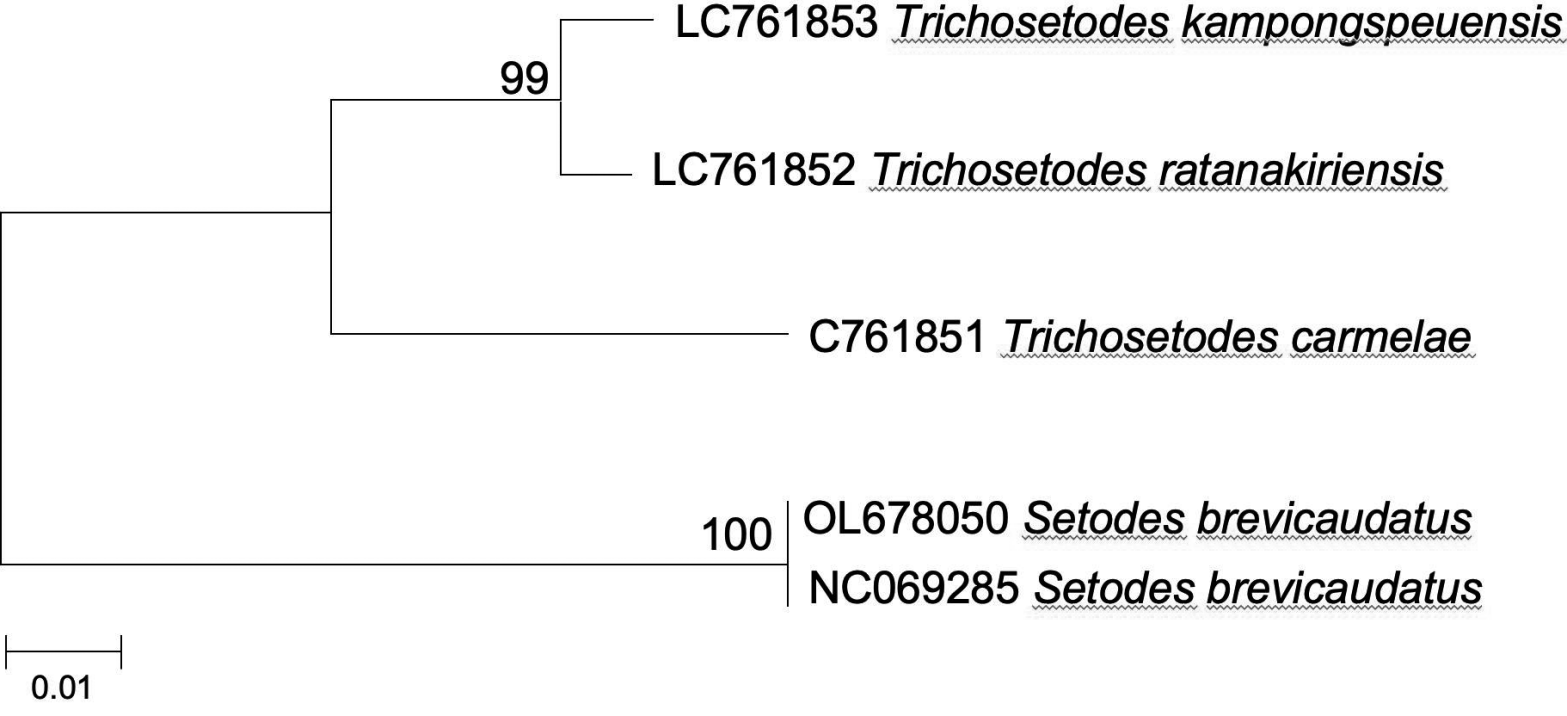
Estimated phylogenetic relationships using the Neighbor-Joining clustering method for *Trichosetodes* spp. based on the mtDNA 16S rRNA region. Sequences of *Setodesbrevicaudatus* were included as outgroups. Each node’s bootstrap value is shown (based on 1000 replicates). The scale bar indicates genetic distance (*p*-distance).

The molecular diversity of the new *Trichosetodes* species was analyzed using the mitochondrial large subunit ribosomal rRNA gene region (16S rRNA). Based on genetic distance (*p*-distance) of this gene fragment, *T.ratanakiriensis* sp. nov. and *T.kampongspeuensis* have a close genetic relationship, whereas *T.carmelae* sp. nov. showed greater genetic divergence from both *T.ratanakiriensis* sp. nov. and *T.kampongspeuensis* (Table 1). The estimated phylogenetic relationships based on the mtDNA 16S rRNA are shown in Fig. 5, where all three *Trichosetodes* sp. nov. were genetically differentiated. The monophyly of *T.ratanakiriensis* sp. nov. and *T.kampongspeuensis* was highly supported by the bootstrap value (NJ BP). In addition, *T.carmelae* sp. nov. was differentiated from the monophyletic clade of *T.ratanakiriensis* sp. nov. and *T.kampongspeuensis* (Fig. 5).

**Table 1.** Genetic distances (*p*-distances) between *Trichosetodes* spp. from the Mekong River basin.

	*T.kampongspeuensis*	*T.ratanakiriensis*
*T.ratanakiriensis*	0.014	
*T.carmelae*	0.070	0.063

## ﻿Discussion

Alongside the previously known *T.kampongspeuensis*, there are now a total of four species of *Trichosetodes* recorded in Cambodia. The three new species are known from a single locality, and such restricted distribution could be attributed to limited regional data availability, thus indicating the need for a comprehensive survey of aquatic invertebrate diversity in the Mekong River basin. Additionally, considering the known distribution of 19 *Trichosetodes* species in Southeast Asia (Fig. 7; Malicky 2010; Oláh 2013; Malicky and Laudee 2017; Morse 2023), such restricted distribution could also be attributed to potentially high endemism of this particular genus in the region (Laudee et al. 2022).

**Figure 6.**
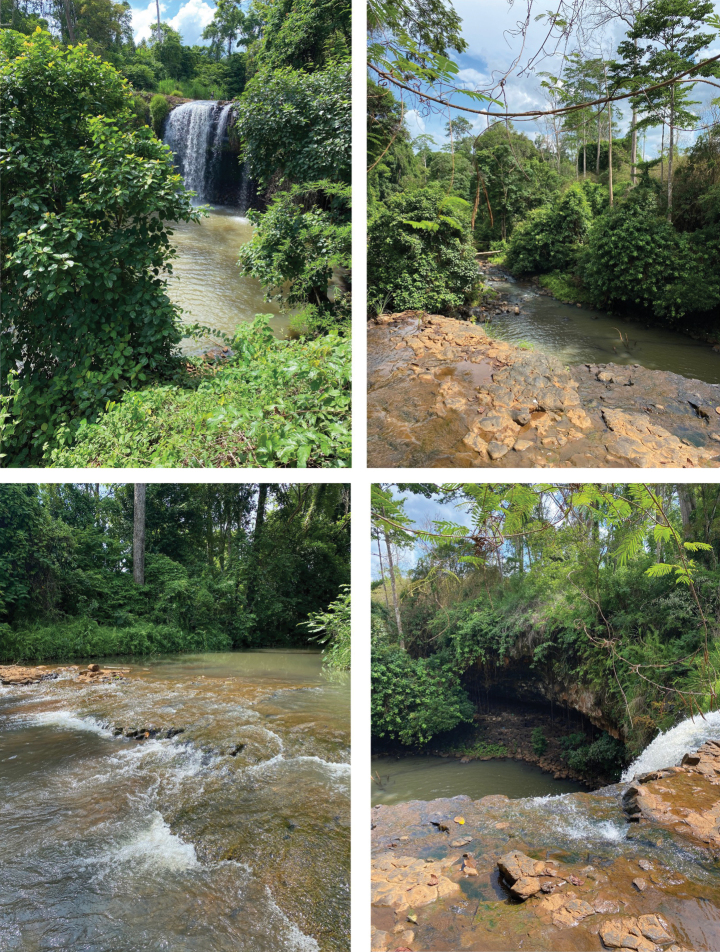
Stream and waterfall at the collection site of *Trichosetodes* spp. in Ratanakiri Province, Cambodia, Mekong River basin.

*Trichosetodescarmelae* sp. nov., *T.katiengensis* sp. nov. and *T.ratanakiriensis* sp. nov. were collected from a waterfall with cover by montane evergreen rainforest in eastern Cambodia. According to the habitat characteristics where they were collected, the three new species of *Trichosetodes* are potentially rhithral species that live in waterfalls and small streams where the substrate is dominated by bedrock, boulders and sand (Fig. 6). Moreover, all four species of *Trichosetodes* recorded from Cambodia were collected from waterfalls (Laudee et al. 2020). Furthermore, *T.asphor* was also collected from a fast-flowing stream (Malicky and Laudee 2017). However, larvae of *T.japonicus* which is widely distributed in East Asia including Honshu and Fukuoka Prefectures (Japan), Korean Peninsula and Far East of Russia were mainly found from middle to lower sections of rivers with slow current (Kawai and Tanida 2018). The larvae and pupae of *T.imperfectus* Ulmer, 1951 from Sumatra, Indonesia were described by Ulmer (1955). Thus, as our collections are based on light trapping and larval stages are still unknown, we cannot exclude the possibility that *T.carmelae*, *T.katiengensis* and *T.ratanakiriensis* inhabit a wide range of habitats, such as larger river sections, lakes and reservoirs like some other Asian species (Kawai and Tanida 2018).

**Figure 7.**
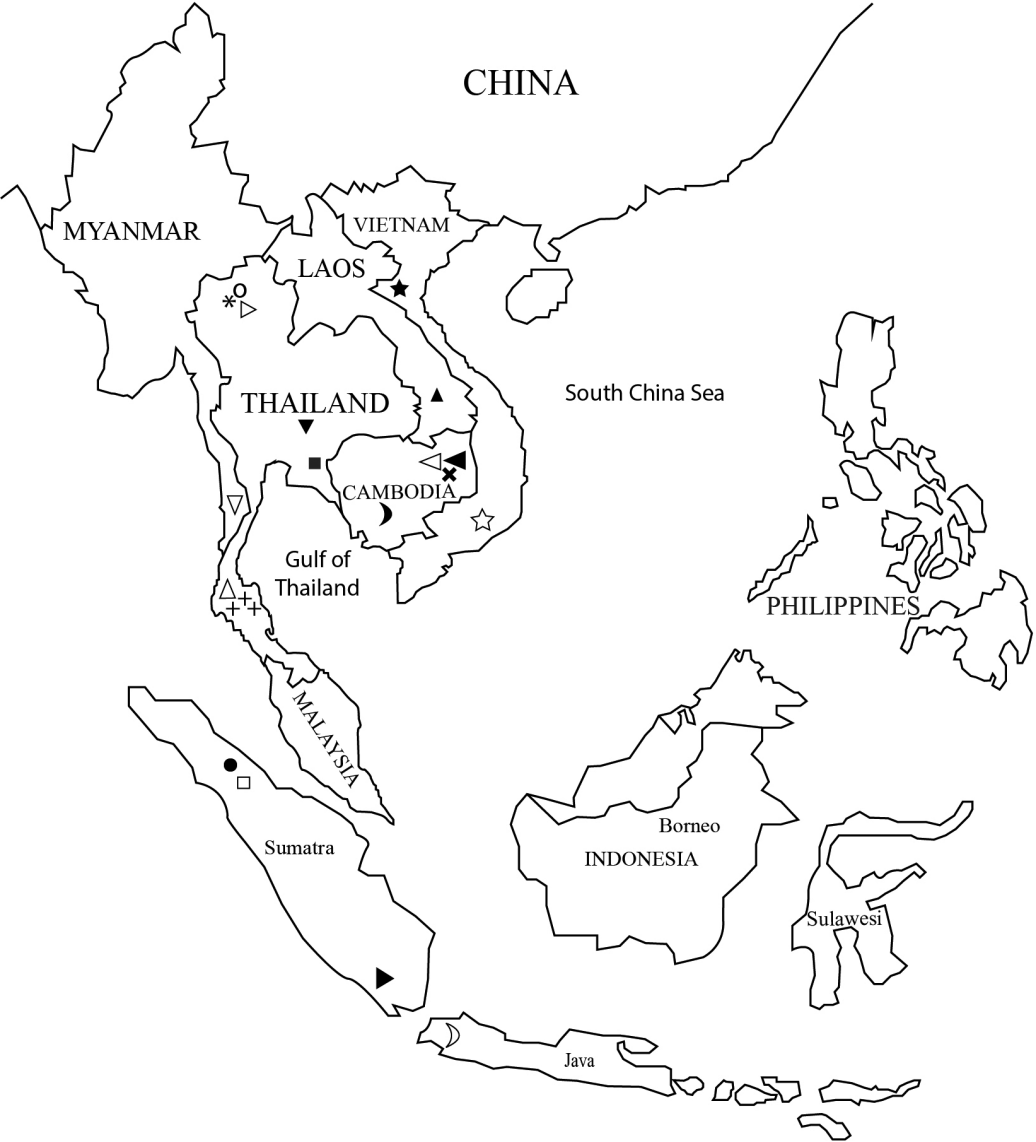
Distribution map of Trichosetodes spp. recorded from Southeast Asia. Abbreviations: **Asterix** = *T.anaksepuluh* Malicky & Chantaramongkol, 1995, **Plus** = *T.sisyphos* Malicky & Taeng-On, 2006, **White up-pointing triangle** = *T.pales* Malicky & Chaibu, 2006, **White circle** = *T.palinurus* Malicky & Chantaramongkol, 2006, **Black circle** = *T.hubertbruckneri* Malicky, 2006, **Black square** = *T.pallas* Malicky & Chantaramongkol, 2006, **White square** = *T.pan* Malicky, 2006, **Black up-pointing triangle** = *T.pandareos* Malicky 2006, **Black down-pointing triangle** = *T.pandion* Malicky & Chantaramongkol, 2006, **White down-pointing triangle** = *T.asphor* Malicky & Laudee, 2017, **Black star** = *T.harmas* Oláh, 2013, **White star** = *T.sotet* Oláh, 2013, **Black moon** = *T.kampongspeuensis* Malicky & Kong, 2020, **White moon** = *T.handschini* Ulmer, 1951, **Black right-pointing triangle** = *T.thienemanni* Ulmer, 1951, **White right-pointing triangle** = *T.anavadya* Schmid, 1987, **Black left-pointing triangle** = *T.carmelae* sp. nov., **White left-pointing triangle** = *T.katiengensis* sp. nov., **Multiplication X** = *T.ratanakiriensis* sp. nov.

The molecular analysis, centered on the mtDNA 16S rRNA region, consistently mirrored the morphological distinctions observed among the newly identified species. While *T.ratanakiriensis* sp. nov. and *T.kampongspeuensis* share similarities in male genitalia characteristics, they exhibit distinct genital morphologies in contrast to *T.carmelae* sp. nov. Concerning the genetic distance calculated from the mtDNA 16S rRNA region, *T.ratanakiriensis* sp. nov. and *T.kampongspeuensis* demonstrated remarkable proximity, with a mere 1.4% divergence. On the contrary, the male genitalia traits of *T.carmelae* sp. nov. markedly differed from those of *T.ratanakiriensis* sp. nov. and *T.kampongspeuensis*, with molecular analysis indicating genetic distances exceeding 6.3%. Our results imply that the mtDNA16S rRNA gene fragment used to infer genetic divergence in Leptoceridae studied proved to be a good tool for supplementing taxonomy and diversity studies of Trichoptera (Takenaka et al. 2023).

## Supplementary Material

XML Treatment for Trichosetodes carmelae

XML Treatment for Trichosetodes katiengensis

XML Treatment for Trichosetodes ratanakiriensis

## ﻿Additional information

### Author ORCIDs

Pongsak Laudee https://orcid.org/0000-0003-3819-7980

Chamroeun Kong https://orcid.org/0000-0002-4141-7270

Masaki Takenaka https://orcid.org/0000-0002-9565-524X

Koji Tojo https://orcid.org/0000-0002-9362-604X

### Data availability

All of the data that support the findings of this study are available in the main text or Supplementary Information.
